# “…Society is, at the end of the day, still going to stigmatize you no matter which way”: A qualitative study of the impact of stigma on social support during unintended pregnancy in early adulthood

**DOI:** 10.1371/journal.pone.0217308

**Published:** 2019-05-23

**Authors:** Heidi Moseson, Moria Mahanaimy, Christine Dehlendorf, Caitlin Gerdts

**Affiliations:** 1 Ibis Reproductive Health, Oakland, California, United States of America; 2 Independent consultant, Ibis Reproductive Health, Cambridge, Massachusetts, United States of America; 3 Department of Family and Community Medicine, University of California San Francisco, San Francisco, California, United States of America; 4 Department of Epidemiology and Biostatistics, University of California San Francisco, San Francisco, California, United States of America; University of Brighton, UNITED KINGDOM

## Abstract

Unintended pregnancy in adolescence and early adulthood is stigmatized in the United States because it deviates from social norms that consider young people’s sexuality as a social problem. While limited, prior research has found that this stigma prevents young people from telling people in their lives about their pregnancies, for fear of judgment or negative reactions. We hypothesized that this selective disclosure of unintended pregnancy due to stigma would reduce the social support available to young pregnant people at a particularly vulnerable time—social support that we know is important for optimal physical and mental health of the young person, and the pregnancy (should they choose to carry to term). To explore this hypothesis, we conducted a qualitative study among young people to understand if and how they experienced stigma in relation to an unintended pregnancy, how this stigma shaped patterns of pregnancy disclosure, the implications for received social support, and participant thoughts on how to alleviate the influence of this stigma on their lives. In in-depth interviews with 25 young people in the San Francisco Bay area who had experienced at least one unintended pregnancy, using a thematic analysis approach, we found that the stigma of unintended pregnancy led participants to selectively disclose the pregnancy to limited people, which in turn cut them off from needed sources of social support. Black and Hispanic women disproportionately described this experience. Participants expressed a desire for programs that would connect young people who had experienced unplanned pregnancy to each other–either via the internet, organized groups through clinical care sites, college or high school campuses, or other forums—as a way to alleviate stigma, share perspectives and lessons learned, and otherwise build emotional and informational support networks for themselves where their usual support had fallen away.

## Introduction

Despite evidence that intention may not capture all aspects of how people view the reality of pregnancy in their lives, whether a pregnancy is intended or not is a central construct that determines if a pregnancy is seen as a positive or negative outcome in the United States, with unintended pregnancy being viewed as problematic from both a public health and societal perspective [1–3]. As a result, these pregnancies are heavily stigmatized, particularly for young people [4, 5]. While no formal definition exists for the stigma attached to unintended pregnancy in early adulthood, this stigma can be conceptualized as a shared (although culturally variable) [6] understanding that unintended pregnancy at a young age is socially unacceptable, and deviates from societal norms that conceive of young people’s sexuality as a “social problem” [7, 8]. This stigma may be primarily experienced as felt and enacted stigma at the interpersonal level, evident in individual interactions with other people[9]. As nearly five percent of reproductive-age women have an unintended pregnancy each year in the United States [10], the existence of this stigma has the potential to impact a large number of individuals.

Unintended pregnancy may be viewed as particularly problematic in adolescence or early adulthood, when society might assume (correctly or not) that a person is ill-equipped to parent a child. Two studies have indeed confirmed the existence of strong stigma toward early unintended pregnancy. (As a note, we define “early” as a pregnancy occurring to a person up until age 25 years. This decision was made in alignment with the fact that unintended pregnancy rates are highest among women in this age range[10], and to capture the experience of unintended pregnancy during the transition to adulthood, a period that is often characterized by much uncertainty.) These studies, however, by including only participants who went on to give birth or by asking about views of *other* people’s unintended pregnancies, may not fully capture the lived experience of unintended pregnancy stigma among young people who choose a range of pregnancy options. The first study, conducted approximately twenty years ago, interviewed over 900 adolescents who gave birth in a Texas hospital between 1993–1996, and found that two in five (39%) reported feeling stigmatized because of their pregnancy [4]. A more recent qualitative study in Alabama conducted among low-income women on their views of unintended pregnancy found that peers view young people who get pregnant unintentionally as “promiscuous”, “irresponsible”, or coming from a “poor upbringing” [11, 12].

This stigma has repercussions. Although the literature on the subject is scarce, findings from prior research have identified an association between stigma and selective disclosure of the unintended pregnancy to one’s social networks. In a 2003 study of middle-class white unmarried women in Southern California, women reported telling few people about their unintended pregnancies, even politically “liberal” members of their social networks, due to strong societal stigma, and consequently, described experiencing isolation and fear [13]. A 2018 study from Norway interviewed thirteen 18–36 year old pregnant women who were undecided about how to resolve a current pregnancy. Participants spoke of similar experiences of feared judgment and, consequently, were also selective in who they chose to tell about their pregnancy. This led participants to describe their decision-making process as a “lonely journey”, and the authors to describe this situation as one of “an existential loneliness” [14]. Beyond selective disclosure, stigma may influence the level of social support people receive during an unintended pregnancy. Although numerous definitions of social support exist, we adopt a definition of social support as the “provision or exchange of emotional, informational, or instrumental resources in response to the perception that others are in need of such aid.”[15] Prior research has indicated that the amount of social support people receive in their everyday lives usually exceeds the amount they expect [16]. Yet, in the case of unintended pregnancy, this may not be the case–in a study of 203 adults that had experienced a recent unintended pregnancy, participants reported receiving *less* social support than expected during their pregnancy, not more[17].

Based on the results of these prior studies, we sought to gain greater insight into the relationship between the experience of stigma and social support during early unintended pregnancy. We hypothesized that, by reducing the number of people aware of the pregnancy, the experience of stigma reduces the social support that individuals receive during their pregnancy–social support that is important for optimal physical and mental health of the young person, and the pregnancy (should they choose to carry to term) [18, 19]. To explore this hypothesis, we conducted a qualitative study among young people to understand how stigma about unintended pregnancy shaped the social support available to them. We specifically focused on the lived experience of stigma related to early unintended pregnancy, if and how stigma shaped patterns of pregnancy disclosure to social network members, the implications for received social support, as well as participant thoughts on how to alleviate the influence of this stigma on their lives.

## Materials and methods

### Study population

We recruited young people in the San Francisco Bay Area between February and December 2016 for interviews regarding their experiences with stigma and social support during unintended pregnancy. An estimated target sample size of 25 individuals was set to reach a minimum level of theoretical sufficiency with regard to participant experiences, and to be consistent with sample sizes in the related literature. Participants were recruited via Craigslist (four posts over the 11-month recruitment period), and via geo-tagged flyers posted a single time in multiple venues in the catchment area. Flier locations included 75 sites on local college campuses, laundromats, coffee shops, gyms, bars, convenience stores, public hospitals, and more. Flier locations were selected to reach a socioeconomically diverse group of young people, who may represent a diverse set of experiences with unintended pregnancy.

Individuals were eligible to participate in the study if they spoke English or Spanish, had experienced at least one unintended pregnancy by age 25 years or younger, and were at least 18 years of age and no more than 30 years of age at the time of the interview. We defined “unintended pregnancy” to mean a pregnancy that was unplanned or unwanted at the time of conception. We acknowledge the limitations of this measure, but utilized it for consistency with the literature. Eligibility was assessed via email or phone call, depending on the mode of contact initiated by the interested individual. If eligible, all individuals participated in an informed consent process, and were offered a $40 gift card for their time. The Institutional Review Board of the University of Anonymous [details omitted for double-blind review process] approved the study.

### Data collection

After obtaining informed consent, participants were interviewed either in-person (at the university, in coffee shops, or in a park) or over the telephone (based on participant preference)by the first author (a cisgender woman, doctoral candidate in Epidemiology with a Masters in Public Health, who had training in qualitative research methods and prior experience conducting interviews). Only the interviewer and interviewee were present for each interview. Participants were told that the interviewer was a graduate student at the university, and that the aim of the study was to better understand “young women’s experiences with unexpected pregnancy.” The semi-structured interview guide asked participants to describe the course of events from the discovery of their unintended pregnancy to its resolution, disclosure of the pregnancy, and any social support available or missing during and after the pregnancy. A brief set of questions captured information on sociodemographic characteristics as well. All participants were interviewed in English by the first-author. All interviews were audio-recorded and professionally transcribed. No field notes were systematically collected, and no repeat interviews were conducted. Approximately 20% of completed interview transcripts (n = 5) then went through a quality assurance process to check and correct for systematic errors in transcribing. Due to not obtaining permission to contact participants in the future, interview transcripts were not sent to participants for their comment and/or correction.

### Analysis

We utilized a thematic analysis approach to analyze these data[20], in which we explored both pre-identified and emerging themes using an iterative coding process during an initial period of familiarization with the data. Our pre-specified themes were drawn from our knowledge of the literature and our hypotheses related to stigma and social support, including desire for social support and experience of disclosure. These themes were analyzed with those themes that inductively emerged in the following process: (1) organizing themes, according to preliminary codes, (2) iteratively refining and expanding codes as necessary, and (3) describing relationships and patterns across codes and interviews. As a first step, the first and second authors developed a preliminary codebook based on high-level themes and topics covered in the interview guide and emerging themes. After all interviews were completed, the first two-authors then performed parallel coding of the same two interview transcripts using the preliminary codebook. The coding process involved reviewing each transcript, line by line, and tagging relevant excerpts with the appropriate code. After the first round of parallel coding, researchers assessed agreement in code application between the two coders, and then updated code definitions and guidelines accordingly. The same two researchers applied this revised codebook in a second round of parallel coding, after which additional updates were made to the codebook. Using this final codebook, the second author completed coding of the remaining interviews. Researchers conducted all coding in Dedoose, an online qualitative software program that facilitates systematic data management, coding, retrieval, and analysis (https://www.dedoose.com/). Once all interviews had been coded, the research team reviewed the transcripts and together identified priority codes and sub-codes that emerged from the data as central to the research question. Code summaries were drafted for each of these selected codes, and this analysis informed the interpretation and synthesis of results. Included quotations are non-identifiably attributed to respondents using the age at the time of the unintended pregnancy, as well as the outcome of that pregnancy (terminated versus continued the pregnancy), and race. No participants reported making an adoption plan. The methods and results of this study are reported in accordance with the COREQ checklist for qualitative research[21].

## Results

### Recruitment

A total of 53 individuals contacted the study team to inquire about participation in the study. Five individuals heard about the study via fliers on their college or university campus, in a health clinic, at a mall, or in a public hospital; one individual found the study via referral from a friend; and the remaining 47 individuals learned about the study via Craigslist. Of these 53 individuals, nine did not respond to eligibility screening questions. Of the 44 individuals who responded to screening questions, six were excluded for being over the age of 30 years, and one declined to participate after learning that the gift card could not be offered in cash. A total of 37 young people self-identified as meeting the eligibility criteria, six of whom never responded after eligibility was confirmed, and an additional six did not show up for the scheduled interview. This resulted in a total of 25 young people who were interviewed for this study, 47% of the individuals that initially expressed interest in participation, and an unknown percentage of individuals who were exposed to study information.

### Participant characteristics and pregnancy outcomes

Study participants represented a range of racial, ethnic and educational backgrounds (Table 1). Seven participants identified as Black, seven as Hispanic or Latinx, seven as White, and four as Asian, and nearly half had completed college or some form of graduate school. All participants identified as women, and thus we refer to participants as “woman/women” throughout, although we acknowledge that some individuals who do not identify as women are capable of pregnancy. Mean age at the time of interview among study participants was 25 years, while mean age at the time of the first unintended pregnancy was 19 years, ranging from 13 to 25 years of age. Among the 25 young women who were interviewed, 42 unintended pregnancies were reported and discussed, of which two ended in miscarriage, 26 in abortion, 12 in live birth, and two women were still pregnant at the time of interview.

**Table 1:** Characteristics of 25 interview participants interviewed in the San Francisco Bay Area in 2016.

Participant characteristics			
Reproductive characteristics		*Mean (range)*	
Age (years)		25 (19–30)	
Number of pregnancies reported			
Total		42	
Mean (per participant)		2 (1–5)	
Age at the first unplanned pregnancy, years		19 (13–25)	
Age at the most recent unplanned pregnancy, years		21 (16–30)	
Number of births		0.5 (0–3)	
Number of abortions		1 (0–2)	
**Race/Ethnicity**		*n*	
Black/African American		7	
Hispanic/Latina		7	
White/Caucasian		7	
Asian		4	
**Education**			
High School		3	
Some College		10	
College		9	
Graduate		3	

### Perceptions of stigma

Interviewees described their perceptions of the views of those around them toward early unintended pregnancy, parenthood and/or abortion. Participants did not always distinguish between whether the stigma was felt to be directed toward the pregnancy itself and what it represented (i.e., having had sex), versus their decision about whether to terminate or continue the pregnancy. In some cases, however, it was clear that participants felt strong expressions of stigma toward the unintended pregnancy itself, regardless of their decision:

*“We penalize women for having abortions, but then we penalize young mothers […] You're stuck in these two hard spots and society is, at the end of the day, still going to stigmatize you no matter which way*.*” (21 years old at time of pregnancy, terminated the pregnancy, Black)*

Other participants described perceiving stigma specifically toward abortion, not to the pregnancy itself. This was common in the sample, and participants felt that the stigma of abortion influenced how they were viewed in their community:

*“I'm from like a really small town in the country*, *so abortion is a bad—that's something evil women do (…) It [abortion] was probably the ultimate shame” (18 years old at the time of pregnancy*, *terminated the pregnancy*, *Hispanic*)

At the same time, other individuals described perceiving judgment directed toward the pregnancy itself, but also toward the decision to *not* abort–both from their social networks and from health care providers. One young woman described her experience:

*“It felt like, also, there’s lot of judgment when younger people get pregnant*, *younger women, there’s a stigma. […] I felt like people judged because I was younger, and then they would say I was unready, and [having a child] is going to make my life worse, I’m going to have it hard or I wouldn’t have my dreams any more, you know?.” (22 years old at time of pregnancy, continued the pregnancy, Asian)*

### Stigma leads to selective disclosure

The shame and stigma that many participants reported perceiving around early unplanned pregnancy influenced their decision to tell others about their pregnancy. While disclosure to the man involved in the pregnancy was nearly universal among study participants, disclosure to family members and/or friends was highly variable, and individuals outside of these groups were rarely informed of the pregnancy. Participants were deliberate in choosing the members of their social network with whom they felt they could share their pregnancy, in most cases only choosing to share with individuals who they felt would not judge them.

Many women described their rationale for not disclosing the pregnancy as based in the hope that if no one knew about the pregnancy, they could more easily avoid judgment or other unfavorable responses. While this theme emerged primarily among women whose pregnancies ended in abortion (as pregnancies that resulted in a child were less easy to hide), some participants who eventually continued the pregnancy reported only disclosing the pregnancy to their parents at a late stage in the pregnancy (e.g. month eight) due to fear of judgment, often rooted in religious norms. One participant discussed this in the context of her pregnancy: “*I was still in college*, *and I know*, *like*, *my parents are really religious*. *Yeah*, *so that was like a big part of it [not telling them]*. *Like I was scared*. *I didn’t even tell them until like a month before I gave birth*.*” (21 years old at the time of pregnancy*, *continued the pregnancy*, *Hispanic)*. A significant number of participants described being apprehensive about sharing their pregnancy with anyone, including their health care providers–even many years later—due to a fear of judgement. Several participants said that they do not report the true number of pregnancies on medical forms, for fear of judgment. One young person described:

*“I think one time I actually wrote like just one pregnancy or like zero, and this was my first*. *And then I later whispered to the nurse and pulled them aside and was like, ‘You know what?’ I whispered and I was like, ‘I had two abortions. I didn’t want to put this down. I didn’t want to put this–I didn’t want to say this for the doctor.’” (16 and 19 years old at the time of pregnancy, terminated both pregnancies, Hispanic)*

### Selective disclosure leads to reduced social support

Most participants in the study expressed not having the social support they wanted during their pregnancy, either from the man involved in the pregnancy, their family, their friends, or all. Participants frequently linked their selective disclosure of the pregnancy to this lack of social support–that because of the stigma associated with unintended pregnancy, they did not tell those closest to them about the pregnancy, and as a direct result, those individuals could not provide the social support they needed during the pregnancy. One woman succinctly described this cycle of stigma, isolation, and lack of support:

*“Even then it’s like I know I’ve gone through [early pregnancy and abortion], and I would love to help someone else, […] someone who has been going through this. But like, (A), I haven’t told anyone else, so it’s not like people know that about me. And then, (B), other people aren’t going to, like, tell people, especially if you’re not like a super close friend. It’s all just kind of very hidden. It’s such a private thing that no one can be a support for each other because no one knows.” (22 years old at the time of pregnancy, terminated the pregnancy*, *Asian)*

Several participants who decided to terminate their pregnancies described this scenario. For instance, one participant reflected:

*"I think the biggest motivating factor that would have made the bigger difference is from the people who didn't know, which I weren't going to tell. And I think that was due to all of the stigma that was placed prior to that. I would have loved to have the support from, say, my father. Like, that would have been awesome. But, no. Like, no." (21 years old at the time of pregnancy, terminated the pregnancy*, *Black)*

Participants who decided to continue the pregnancy and parent described a similar pattern of selective disclosure leading to missed support *during* the pregnancy, although support that was lacking *after* the birth seemed to weigh more heavily on their experiences. Many reported that stigma toward early parenthood stymied the support they think they might otherwise have received. One woman reported:

*“But it’s been a struggle ever since he was born, because no father, no help. Because, like, my family, they don’t believe in having children out of wedlock due to their religion. So, like, my mom, she would help when she could, but everybody’s busy. […] I really didn’t have a lot of family support or friend support.” (20 years old at the time of pregnancy, continued the pregnancy, Black)*.

Other participants highlighted not just the lack of support from friends and family, but also structural institutional support that was lacking for young, new parents. One participant spoke of the reinforcing aspect of these gaps in support–because she did not have supportive housing close to campus, she needed additional childcare and had to commute long hours, which further isolated her by preventing her from having any time to connect with peers:

*“I mean, […] like if the university had family housing. I had nowhere to live. [My boyfriend] was supporting me. […] And because I was under so much pressure with studying and commuting, it was like I’d go to class, study, commute, and not have time to, like, connect with any of the other students.” (20 years old at the time of pregnancy, continued the pregnancy*, *White)*

It bears mention that examining participant experiences through the lens of race indicated a pattern of stronger experiences of stigma toward early pregnancy and abortion for Asian, Black, and Hispanic women, versus White women. Perhaps of most consequence, among those who explicitly mentioned negative cultural perceptions of pregnancy and/or abortion leading to a lack of support, this was disproportionately (and in absolute terms) more common among Black women than among White women. One young Black woman described this link between judgment from others and the support she received for her two pregnancies:

*“I mean, it’s hard enough finding someone who will be a financial support to you, a place to stay when you need it. So, that is like–because that’s morals. That’s a moral humungous debate because it’s [abortion] literally life or death. So, even the people that you think will be your friend and have your back might not, like might actually be like, ‘I don’t want to be your friend because I wouldn’t be friends with somebody who does that’ [abortion].” (23 and 25 years old at the time of pregnancies, terminated both pregnancies*, *Black)*

### Addressing the isolation of unintended pregnancy

In the process of reflecting on their own pregnancies, participants shared their perspectives on what could be done to counter the isolating influence of stigma, and to increase support for young people faced with an unintended pregnancy. Above all else, participants described wanting connection to other people who had also experienced an unintended pregnancy at a young age—to be able to ask questions of someone who had been in their position, who would not judge them. Nearly all participants felt that being connected in this way would reduce the isolation and stigma that they felt and could provide them some of the answers and support they needed at that time. Although the specific questions and types of support that each woman needed varied–from guidance in making an abortion appointment to help with childcare to just needing someone to talk to–nearly all participants expressed that being able to ask others about their experiences would have been of tremendous comfort and practical help. One young woman described the desire for this support as follows:

*“Maybe having someone who had gone through the same thing talk to me about it, like, at a similar age. […] Something to just let me know that, like, I can become the person that I am today, someone like me. If there was someone like me now, working at the clinic, like, if they had someone that you could just talk to, to tell you like the things that you don’t even know you want to hear. […] Like that life will go on, and it’s not the end of the world. [No] one ever said that to me.” (13 years old and 22 years old at the time of her pregnancies, terminated both pregnancies*, *White)*

Other participants described a potential group setting for young, pregnant people to provide emotional and informational support for each other–an idea that came up in several interviews about the support women felt they had or did not have during pregnancy. One particular participant highlighted the idea that continuity of emotional support during and after the pregnancy would have been helpful for her.

*“I feel like emotional support […] Yeah, that would’ve been helpful. Maybe, I don’t know, an emotional–like a session to talk about it during the pregnancy and then also checking in after to make–[to ask] how are you doing? How are you feeling about it? But genuinely actually caring about another.” (15 years old and 17 years old at the time of her pregnancies, terminated both pregnancies, Hispanic)*.

A small number of women reported finding such a connection to other young pregnant women during their pregnancy–whether online, in formal support groups, or from their social networks—and the positive, empowering impact it had on their experience of unintended pregnancy. Those who described online support groups commented on how the frequent ability to connect with others was beneficial. One participant described: “The website online was, like, my best friend throughout the entire process in trying to ensure that—like, normalizing what I was going through. Like, "Oh, this is what everyone else's experience is. Oh, I'm not being overdramatic. This is really how people feel." Yeah.” *(21 years old at the time of pregnancy*, *terminated the pregnancy*, *Black)* For other participants, in-person support groups were vital. One woman talked at length about the validation and encouragement and practical tips for pregnancy and parenting that she received through a support group for young pregnant women organized by the Black Infant Health Program:

*“And in the groups, you know, we talk about our goals during our pregnancy, support. It just–it's a really good group. […] just being, you know, unified as women, you know–it means a lot*.*” (23 years old at time of pregnancy, continued the pregnancy, Black)*

## Discussion

In in-depth interviews with 25 young women in the San Francisco Bay area who had experienced at least one unintended pregnancy, participants reported strong perceptions of stigma toward early unintended pregnancy from their social networks that prevented them from disclosing the pregnancy to family members and friends with whom they were otherwise close, for fear of judgment or negative reactions. This selective disclosure led to social isolation of the young women, cutting them off from their usual trusted sources of information and support in a vulnerable time. Participants presented their own ideas for how to alleviate the stigma and isolation of unintended pregnancy at a young age. Almost universally, participants expressed a desire for connection to other young people who had also experienced unintended pregnancy as a way to alleviate stigma, share perspectives and lessons learned, and otherwise build emotional and informational support networks for themselves where their usual support had fallen away.

The health implications of these feelings of social isolation during pregnancy may be of consequence. A seminal 1988 review of scientific research suggested that the health effects of loneliness and social isolation in general might be equivalent to those resulting from high blood pressure, obesity, lack of exercise and smoking [22]. More recent research has confirmed and expanded on these findings [23–28]. Social isolation is associated with increased levels of stress hormones and inflammation throughout the body, and further, particularly when there is disruption to important social-emotional relationships, may negatively alter neuroendocrine processes in the brain [29]. Additionally, recent research suggests that the negative effects of social isolation may be most pronounced among younger individuals [28]. These physiological relationships tied to social isolation feel particularly consequential in light of the racial differences identified in this study in the degree to which stigma was felt, and its impact on social support—with Black and Hispanic women in particular more likely to describe stronger stigma tied to reduction in support received. Given what we know about racial disparities in pregnancy and birth outcomes[e.g., [30, 31]], and their ties to chronic and acute stress [32, 33], it is possible that some aspect of this pathway may be mediated by or partially determined by experiences of stigma, and in turn, ties to social support [34]. Further research could explore how this stigma may relate to or be caused by racism, and particularly on how, on a societal level, fertility is valued differentially by race/ethnicity. [35, 36]

There is evidence to suggest these same pathways between social isolation and adverse health outcomes may be at play among pregnant women in particular. A 2007 study of nearly 900 women followed from the first trimester of pregnancy until after birth found that women with low levels of perceived social support reported increased depressive symptoms and reduced quality of life as compared to women reporting higher levels of social support, and that the infants that they gave birth to were more likely to be small and had a markedly lower birth weight (by an average of 200g) than did infants born to women reporting higher levels of social support [37]. Another survey of approximately 15,000 mothers that delivered in Australia in 2002–2003 found that feelings of social exclusion independently predicted maternal depressive symptoms, as well as maternal responsiveness to the infant [38]. In short, beyond the negative health effects of social isolation known to exist broadly, there is evidence to suggest that these factors may also operate in the acute pregnancy phase, with negative health consequences for both the pregnant person and the infant, should the pregnancy go to term.

To counteract the stigma and isolation experienced during their pregnancies, participants expressed a desire for connection to other young people who had experienced an unintended pregnancy at a young age and resolved it in a similar way (i.e., abortion versus parenthood). Social support interventions have been designed and tested in a range of health settings for outcomes spanning cancer, weight loss, loneliness, mortality, and more [39]. In considering whether an intervention designed to connect young people in this way might be promising, we looked to the literature for similar examples of social interventions among pregnant individuals in particular. One particularly widespread social intervention, Centering Pregnancy[40], was designed to improve pregnancy and birth outcomes for pregnant people of all ages. This program brings together pregnant people (and their partners, when relevant) to participate in group-based prenatal care with others who are within several weeks of the same gestational age. Numerous studies have found this program to be associated with positive physical and emotional outcomes for those who participate [e.g., [18, 41]]. Similarly, a recent Cochrane review of diverse psychosocial and psychological interventions administered as a part of prenatal care reduced the number of pregnant people who developed postpartum depression [42]. Among the most promising of these social interventions tested was a peer-based telephone support system, a model that is perhaps readily adaptable to the unintended pregnancy context. Other research among young, adolescent parents has identified positive responses to social support interventions in terms of mental and psychological wellbeing, as well as parenting ability [43–45]. With direct implications for racial disparities in pregnancy and birth outcomes, a 2010 study of 222 clients enrolled in a pre- and inter-conception care case management program found that social and behavioral interventions designed to build resilience among high-risk women showed promising results related to reducing infant mortality and low birth-weight births, among other outcomes [46]. Future research will explore possible models for a socially-based, peer-connection intervention to reduce isolation and improve the emotional and physical well-being of young people experiencing an unintended pregnancy.

While this qualitative analysis offers new insights into the relationship between stigma and social support during early unintended pregnancy, it is limited by a number of factors. Firstly, the narrow geographic scope of this study—with nearly all participants residing in the San Francisco Bay Area of California–may limit generalizability. The views expressed by participants may differ from the experiences of stigma felt by other young pregnant people in the United States. The stigma felt and experienced by our participants may be a lower estimate of the stigma felt by young people living in more conservative areas of the country, where social norms against early unintended pregnancy may be more severe. However, recent work among women who underwent abortion in California found that mean scores on the Individual Level Abortion Stigma Scale (ILASS) were comparable, and slightly higher, to what had previously been obtained in a national study [47]–consistent with our findings that stigma was strong even in a more liberal region of the United States. Finally, as these results were collected as part of exploratory research, there may be additional themes and experiences that we did not capture. As we did not seek permission to contact study participants after data collection was complete, we were unable to validate the final themes with participants (member check)–a method that could have increased the internal validity of study findings. This study is strengthened, however, by the recency of participants’ pregnancy experiences, which should contribute to their ability to accurately recall their perspectives and needs at the time of the pregnancy, by the racial and ethnic diversity of participants, and by the novel focus on the link between stigma, selective disclosure, and social support received. Significantly, these findings provide consistent recommendations from young people themselves on how to address the detrimental pathways associated with stigma, social support, and unintended pregnancy.

## Conclusions

Our findings on the existence of stigma toward unintended pregnancy, and of the resulting selective disclosure of the pregnancy are consistent with prior literature, but the link between selective disclosure and reduced social support is novel, as are the suggested methods of addressing this stigma and resulting isolation. We find that the stigma experienced during unplanned pregnancy in adolescence and early adulthood leads to isolation of the young person that is both detrimental and modifiable, and seemingly exacerbated for young people of color. Stigma around unintended pregnancy indeed caused participants to keep their pregnancies fairly secret, and because of this, young people did not ask for or receive needed support. This secrecy further propagated stigma, causing other young pregnant people to feel isolated and stigmatized and preventing them from being able to reach out to others with shared experiences to share lessons learned, words of encouragement, or other support. Moving forward, researchers should explore ways of connecting young people who have experienced unplanned pregnancy to each other–either via the internet, via organized groups through clinical care sites, via college or high school campuses, or other forums.

## Supporting information

S1 File Semi-structured interview guide. This guide contains the questions and probes used by the interviewer in guiding the in-depth interviews with study participants.
(PDF)
