# Supplementary material for: “…Society is, at the end of the day, still going to stigmatize you no matter which way”: A qualitative study of the impact of stigma on social support during unintended pregnancy in early adulthood
Source: PLoS One. 2019 May 23;14(5):e0217308. doi: 10.1371/journal.pone.0217308 (PMC6532899; doi:10.1371/journal.pone.0217308)
Supplement: S1 File — This guide contains the questions and probes used by the interviewer in guiding the in-depth interviews with study participants. (PDF) [file pone.0217308.s001.pdf]

Semi-Structured Interview Guide

Introductory Script:
Thank you so much for taking the time to meet today. My name is Heidi, and I am a graduate student at the University of California, San Francisco. The goal of this study is to better understand the social support available to women when deciding whether or not to keep an unintended pregnancy, and whether or not we can improve this support to make the decision process easier. We hope that information from this study will help us provide better counseling and support services to other young, pregnant women. The interview should last about 45-60 minutes, but if you ever feel uncomfortable with any questions, we can just move on to the next. Just let me know. If you need any question clarified, just stop me and ask me. Please take your time answering any of these questions - I don't expect you to have an answer right away. Do you have any questions now? If not, let's begin.

- **Do you mind telling me your age now?**
- **Age when you discovered your unintended pregnancy?**
- **Education?**
- **Ethnicity?**
- **Zip Code?**
- **Where you heard about study?**

1. Walk me through what happened from the time you discovered your pregnancy until it ended.

Probes:

- Were you on any birth control?
- Did you talk to anyone about it?
- Who? When? Why?
- ▪ How did they respond?
- ▪ If woman mentions her partner, ask: Tell me more about the involvement of your partner. Do you think more or less (or different) involvement from him would have helped you make your decision or feel more confident in your decision? If yes, how? If no, why not?
    - Provide instrumental support? Emotional? Other?
- *If reported miscarriage*: probe to assess whether it was induced by medication abortion.
- Where/how did you learn about your options for keeping or terminating the pregnancy?
- How confident were you in your decision?

2. How did you feel about the social support available to you while you made your decision?

Probe:

- Was the opinion of anyone else important to you? If yes, who?
- Did you feel that you wanted more support? If yes, in what form? From who? When? If no, why not?
- Did anyone talk to you about abortion? If so – how? Did you get a sense that they had opinions (moral or emotional or otherwise) about abortion as an option?

- Do you and your friends ever talk about abortion? If so, in what context? How?

3. Was there any specific information that you wanted to know about your options?

Probes:

- For instance, any information on the costs of various options (adoption, abortion, parenthood), what the procedures entailed, where you could get them, adoption options, connections to women who had been in your position before, etc?
- Were you interested in how it would affect your chances of: finishing high school or college; being on welfare; depression; relationship future (marriage)?
- If yes, where did you go to find this information? (nurse, partner, friend, parent, internet, other?)
- What did you know about abortion? Adoption? Prenatal care?

4. Tell me about your interactions with any health care providers after you found out you were pregnant.

- What could s/he have done to make the decision-process easier for you?
- Is there anything s/he did that was very helpful? Very unhelpful?

5. Can you think of any way that making your decision could have been made easier, or helped you to be more satisfied?

Probes:

- If yes: tell me more about what that would look like.
- How would you react to a decision-support tool provided to you when you got your positive pregnancy test – something that would provide information to you on a variety of questions related to keeping or terminating a pregnancy in early adulthood, and that might have a social aspect – connecting you to other women (anonymously) in the same position?
- What format might be most useful/user-friendly: informational website, interactive iPad application, telephone hotline, referral to counseling service, or other?

6. At the time you got pregnant, how often did you feel that there were people you could turn to?

- (sometimes, always, rarely, never)
- When you answer this, are you thinking financially? Emotionally? Other
- How did you feel about this now?

Closing Script: That is all of the questions that I have on my end. Is there anything else that you would like to talk about? Do you have any questions, about what we discussed today, or the study more generally? *If no, or after addressing further questions*: I want to thank you again for taking the time to talk with me today. We are so grateful to you for your time. If you have any other questions that come up, please feel free to email or call us at any time using the contact information we provided for you.
